# Single-Walled Carbon Nanotubes as Optical Transducers for Nanobiosensors In Vivo

**DOI:** 10.1021/acsnano.4c13076

**Published:** 2024-12-19

**Authors:** Zachary Cohen, Ryan M. Williams

**Affiliations:** † Department of Biomedical Engineering, The City College of New York, New York, New York 10031, United States; ‡ PhD Program in Chemistry, The Graduate Center of The City University of New York, New York, New York 10016, United States

**Keywords:** SWCNT, in vivo, mice, implants, nanosensor, biosensor, near-infrared fluorescence, in planta, point-of-care

## Abstract

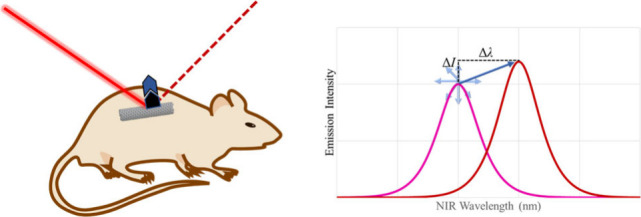

Semiconducting single-walled carbon nanotubes (SWCNTs) may serve as signal transducers for nanobiosensors. Recent studies have developed innovative methods of engineering molecularly specific sensors, while others have devised methods of deploying such sensors within live animals and plants. These advances may potentiate the use of implantable, noninvasive biosensors for continuous drug, disease, and contaminant monitoring based on the optical properties of single-walled carbon nanotubes (SWCNTs). Such tools have substantial potential to improve disease diagnostics, prognosis, drug safety, therapeutic response, and patient compliance. Outside of clinical applications, such sensors also have substantial potential in environmental monitoring or as research tools in the lab. However, substantial work remains to be done to realize these goals through further advances in materials science and engineering. Here, we review the current landscape of quantitative SWCNT-based optical biosensors that have been deployed in living plants and animals. Specifically, we focused this review on methods that have been developed to deploy SWCNT-based sensors in vivo as well as analytes that have been detected by SWCNTs in vivo. Finally, we evaluated potential future directions to take advantage of the promise outlined here toward field-deployable or implantable use in patients.

## Introduction

### The Rationale for Implantable Biosensors

Implantable biosensors have the potential to drastically improve disease treatment, environmental remediation, and the study of animal disease models. Fully realized, such devices would not only facilitate rapid, accurate point-of-care diagnosis, but also enable continuous monitoring of diseases or relevant molecules of interest in plants.^1−3^ The recent COVID-19 pandemic has highlighted the importance of rapid and reliable diagnostics not based on expensive and time-consuming laboratory procedures, which are often slower than the rate of transmission.^4^ Such an expedited diagnosis would also improve the treatment of cancer, which is more successful the earlier it begins.^5^ There is further evidence to suggest that a rapid diagnostic could partially alleviate the socioeconomic disparities in patient outcomes and five-year survival rates.^6^ It is also clear that environmental pollutants, pesticides, and other contaminants have detrimental effects not only on the population but on the health of crops and wild plants.^2^ Furthermore, in some diseases, current lab techniques are orders of magnitude less sensitive than they would need to be to detect biomarkers in blood or extracted plant tissues.^1^

Implantable biosensors would also enable doctors to tailor drug regimens to better suit patients’ individual pharmacokinetics and clearance rates, sometimes known to vary by an order of magnitude between individuals of the same weight.^7^ While multivariate approaches considering a broader range of patient characteristics can result in improved prescriptions, these models may still overlook some important differences.^8^ This presents a challenge especially in the prescription of anticancer drugs, whose narrow therapeutic windows cannot be easily identified on a per-patient basis in the absence of viable pharmacokinetic profiling tools.^9^ Patient genotyping is also not able to entirely resolve all individual differences;^10^ a meta-review of personalized psychiatric treatment studies 2000–2021 found pharmacogenetics to be somewhat variable in its success, while pharmacokinetic analyses could improve dosing efficiency and safety.^11^

The real-world benefits of continuous monitoring have already been demonstrated by the glucometer, a device now seen as essential to the management of diabetes,^12^ a disease affecting 13.0% of adults in the United States based on estimates from 2018.^13^ Self-testing glucose strips, though in widespread use, are less effective than continuous glucose monitoring (CGM) because they depend on an uncomfortable blood sampling process that necessitates infrequent measurements while also discouraging patient compliance.^14^ CGM results in better outcomes for patients because it more accurately monitors typical patterns of glucose levels throughout the day, patient response to treatment regimens, and the risks of hyper- and hypoglycemia, all of which allow for the design and implementation of better treatment plans.^15^ Further advances may allow generalization of this approach to diagnose and treat a wider variety of diseases.

Beyond human and clinical diagnostics, there is substantial potential for improving crop efficiency and health.^3^ Such applications have specific considerations as compared to human or mammalian applications, which must be addressed in design and screening.^16^ To this end, there has been substantial progress in developing technologies, with a focus on nanotechnologies, for environmental monitoring.^2,17,18^ Such technologies have been demonstrated that remotely monitor and report the status of various pesticides, pathogens, or other contaminants through electronic means.^19^ The continued development of such technologies has the potential to reduce food costs, reduce application of harmful pesticides, and combat climate change-related threats to global health.^2^

### Fundamental Nanobiosensor Characteristics

A sensor is composed of a transducer and a recognition element, in addition to tools to measure signals from the transducer. Interaction of the analyte with the recognition element modulates or triggers the transducer’s signal, such that that signal may be correlated to the analyte quantitatively. When a sensor’s transducer is on the nanoscale (i.e., less than 100 nm in any dimension), it is termed a nanosensor; nanobiosensors are nanosensors for biological analytes.^20^ Sensors should not be conflated with contrast or imaging agents, which are used to visualize and determine the location of diseases. A change in transducer signal, as measured by the detector, in response to the analyte presence, absence, and amount, is the major differentiating factor. Bioderived recognition elements like antibodies^21,22^ and enzymes^23−25^ are commonly employed for their high specificity and biocompatibility, though synthetic recognition elements such as aptamers,^26^ imprinted polymers,^27^ and others^28^ are fields of expanding interest.^29^

Many sensors can be categorized as either optical or electrochemical, using light- or electricity-based signaling, respectively. Electrochemical sensors use electrodes or transistors as their transducers, thereby relating the analyte concentration to measurable changes in electric current. Despite the success of CGM, in vivo or on-board electrochemical biosensors remain difficult to translate to the clinic.^30−33^ The requirement for a physical connection to a power source places an intrinsic limit on the comfort and longevity of such devices, as demonstrated by the two-week lifespan of CGM devices.^34^ Optical sensors, in contrast, facilitate measurement using an externally powered instrument (commonly via fluorescence) and may therefore enable in vivo sensing.^35^ However, the implant depth of an optical sensor is limited by the signal’s ability to penetrate tissue.

### The Use of Single-Walled Carbon Nanotubes as Sensor Transducers

Semiconducting single-walled carbon nanotubes (SWCNTs), cylindrical allotropes of carbon, have garnered considerable research interest since their 1991 discovery for their exceptional mechanical, electrical, and optical properties, as opposed to other materials such as multiwalled carbon nanotubes (MWCNTs), which are not inherently fluorescent in the near-infrared region.^36^ Other carbon nanomaterials, such as graphene and carbon/graphene quantum dots, do have intrinsic photoluminescence,^37−40^ though they have not been evaluated as extensively as quantitative in vivo molecular sensors.^41^ Graphene and MWCNTs are commonly used as transducers for electrochemical sensors, which have found some translation to in vivo sensing in that context.^42,43^ Carbon and graphene quantum dots are more often used as optical imaging agents instead of quantitative biosensors.^44−46^

**Figure 1:**
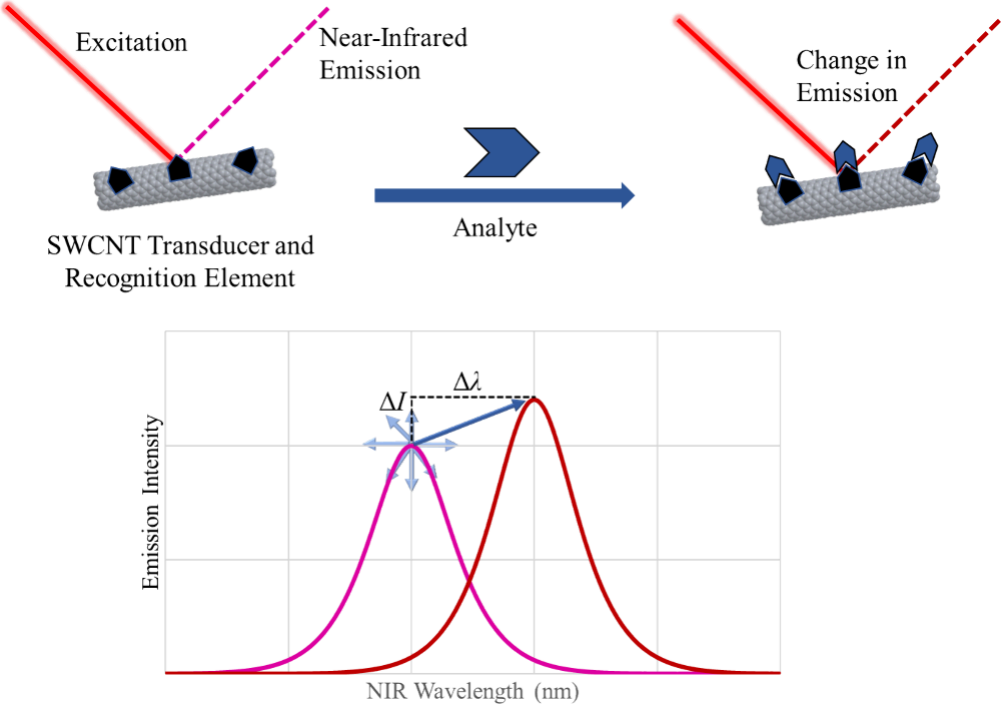
Response of a SWCNT-based fluorescence sensor. Interaction of the analyte with the SWCNT transducer modulates fluorescence upon excitation. In the above example, analyte binding to the sensor induces an SWCNT increase in intensity and red-shifting in center wavelength (to the right). However, the magnitude and change in energy of fluorescence modulation is specifically dependent on the interactions of the analyte, binding element, and SWCNT transducer, as well as local environment, and thus may manifest as any combination of a modulation of intensity (ΔI) or modulation of center wavelength (Δλ).

Fluorescence is inherently sensitive to the microenvironment of the fluorophore; changes in that microenvironment provoke measurable, quantifiable changes in the intensity or characteristic wavelength of fluorescence emission.^47^ What sets semiconducting SWCNTs apart, as a function of their electronic bandgap structure, is that SWCNT photoluminescence is not degraded by excitation, preventing photobleaching,^48^ they fluoresce in the near-infrared tissue-transparent window, and they exhibit a large Stoke’s shift with a narrow emission peak.^49^ Optical SWCNT-based sensors are typically constructed to induce analyte interaction with the nanotube surface through corona-phase interaction, adsorption, or specific molecular recognition with biomolecules. This induces a change in fluorescence emission intensity and/or center wavelength (Figure 1), thus allowing for quantitative detection by evaluating the magnitude of fluorescence response. Further, SWCNT-based optical sensors have the potential to be multiplexed, as the family of semiconducting SWCNTs includes a diverse array of structural isomers, some of which absorb light at similar wavelengths while emitting light at appreciably dissimilar wavelengths.^35,50^ Another advantage is that their large Stokes shifts of approximately 0.52 ± 0.36 EV (915 ± 350 nm) enables greater signal-to-noise ratios by minimizing interference from excitation/emission overlap.^50^ Despite SWCNTs having a relatively low quantum yield compared to organic fluorophores, their in vivo capabilities are strong due to the tissue penetration of SWCNT near-infrared emission, whereas organic fluorophores exhibit visible emission that has less penetration through tissue.^51^

### Types of SWCNT-Based Sensor Designs

SWCNT-based optical nanobiosensors require the nanotube itself to be suspended in solution to serve as a fluorescent transducer, as bundled or solid-phase nanotubes exhibit no fluorescence. Because the hydrophobicity of as-synthesized SWCNTs drives them to form self-quenching aggregates in water, they must be aqueously dispersed for applications in biological systems. This can be achieved without chemical modification of the SWCNTs by via probe-tip sonication with a suitable dispersant.^52,53^ Commonly used surfactants include sodium deoxycholate, (SDC/DOC)^54^ sodium dodecylbenzenesulfonate (SDBS), sodium dodecyl sulfate (SDS),^55^ sodium cholate (SC),^56,57^ Tween80,^58^ pluronics/poloxamers,^59^ cetyltrimethylammonium bromide (CTAB),^60^ and others.^61,62^ Certain biological molecules have also been used to disperse SWCNTs in solution, including peptides^63−65^ proteins,^65−67^ lipids,^68^ and polysaccharides.^69^ Others have used highly modular synthetic polymers.^70,71^ Single-stranded DNA oligonucleotides are also commonly used because they are stable, biocompatible, and commercially available in practically any desired sequence with optional addition of functional groups or dyes.^29,72−75,70^

**Figure 2:**
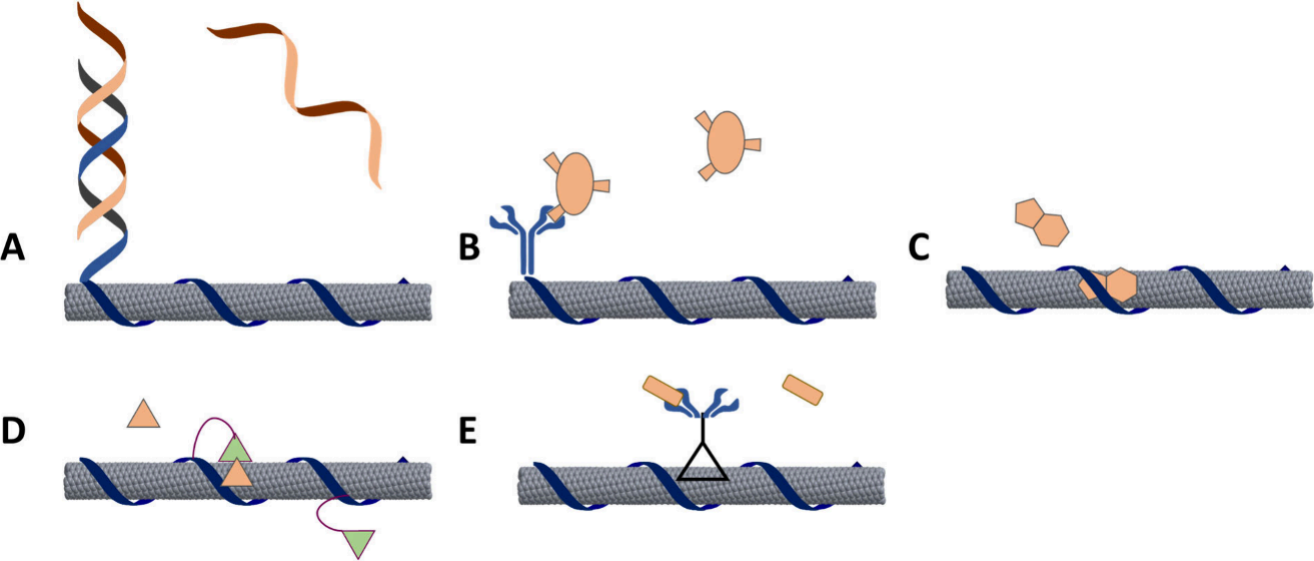
Common SWCNT functionalization methods with demonstrated functionality in vivo. In each panel, the gray tube represents a SWCNT transducer, the blue elements represent SWCNT functionalization methods, and the orange elements represent various analytes of interest. A) A method for complementary nucleic acid base pair detection. Dark blue ssDNA stabilizes SWCNTs in solution and light blue is complementary to the sequence of interest (orange). B) A method for protein antigen detection. Dark blue ssDNA stabilizes SWCNTs in solution and a light blue antibody is conjugated to it, which detects the analyte of interest (orange). C) A method of small molecule (orange) detection based on its interaction with dark blue ssDNA used to stabilize SWCNTs. The ssDNA may have some intrinsic or screened affinity for the analyte of interest. D) CoPhMoRe-based detection of a small molecule analyte (orange), wherein dark blue represents a screened polymer or ssDNA that has creates binding pockets for the analyte. This may be enhanced by target templating, using conjugated target (purple). E) Integration of sp^3^ carbon functionalization, termed organic color centers (OCCs) or quantum defects, which may impart selectivity for an analyte itself or, in this example, after conjugation with an antibody fragment.

To develop nanobiosensors specific for certain biomolecules, many approaches have further functionalized well-dispersed SWCNT complexes with biorecognition elements complementary to their target analytes (Figure 2). Antibodies,^21^ enzymes,^76^ aptamers,^77−79^ and complementary nucleic acids^80^ have all been used in this capacity.^29^ Analyte binding elements promote interactions with the target, imparting sensor specificity. An additional polymer or protein coating can be applied after the recognition element to occlude nonspecific adsorption to the sensor, thereby passivating it to interferants and biofouling, which are particularly detrimental in vivo.^3,21,22,29,81^

Successful, specific analyte detection is also possible using direct recognition via the SWCNT surface coating.^28^ Direct recognition through the surface coating may be possible for any analyte, including several with an innate affinity for the SWCNT surface, such as redox-active or hyperconjugated molecules. This approach circumvents the cost and stability issues inherent to biologically derived analyte complements (antibodies, enzymes) but often necessitates more screening or computational processing (commonly machine learning) to ensure specificity.^82−85^

Apart from targeting biomarkers with inherent affinity for the SWCNT complex, SWCNT sensors have also been developed using the screening-based approach Corona Phase Molecular Recognition (CoPhMoRe), wherein nanobiosensor selectivity arises from the surfactant coronas formed upon nanotube solvation.^28,86^ While the approach has existed since at least 2010,^87^ the term CoPhMoRe was coined in 2013.^28^ Though antibody or aptamer screening often occurs separately from SWCNT sensor construction, CoPhMoRe screening takes advantage of the interactions of SWCNTs with constituents of a small synthetic polymer or DNA library.

Surface coatings for such sensors can be empirically informed or based on computational models.^88−90^ Alternatively, further affinity may be engineered via incorporation of an analyte template, an approach analogous to molecularly imprinted polymers.^91^ Many such direct-detect nanobiosensors employ oligonucleotides for their stability and biocompatibility, but other polymer surfactants (polyethylene glycol, dextran, etc.) are also commonly used.^92^ These approaches have been demonstrated for a diverse array of analytes, including nitric oxide (NO),^87^ riboflavin,^28^ L-thyroxine, estradiol, dopamine,^86^ fibrinogen,^93^ insulin,^94^ hormones,^91^ and cytokines.^95^

A particularly exciting direction of SWCNT sensor development has been in the application of organic color centers (OCCs), also called quantum defects.^96−99^ OCCs incorporate covalent modification at sp^3^ sites on the sidewall of sp^2^ carbon atoms, creating an additional red-shifted photoluminescence peak with increased relative quantum yield.^97,99−101^ OCCs are then commonly wrapped with ssDNA or other polymers and have been used extensively over the past decade for sensing applications, including for dopamine,^96^ pH,^102^ and even ovarian cancer with the assistance of ML-based spectral fingerprinting.^83^ Further, direct conjugation to the defect site is possible, with a recent example of antibody fragment covalent modification to detect the inflammatory cytokine IL-6.^103^ There is one example of OCC sensors used in vivo to detect lysosomal pH after intratumoral injection into ovarian cancer xenograft models.^104^ However, given the promise of this technology, it is likely that the use of OCCs will continue to be deployed in live plants and animals.

#### Methods for SWCNT Sensor Deployment In Vivo

Many impressive methods have been developed for analysis of tissues and fluid samples using SWCNT-based sensors ex vivo.^21,105^ These include detection of glucose and dopamine in mouse brain slices,^106,107^ as well as the estrogen receptor in patient breast cancer biopsies.^108^ Additionally, many applications of optical SWCNT sensors in live mammalian cells or in single-celled organisms such as bacteria have been demonstrated.^52,109−111^ There have also been several studies investigating SWCNT imaging in living worms (*C. elegans*)^112^ and rodents without a molecular sensing component.^52,113−115^ An early theoretical and modeling-based study predicted the feasibility of developing SWCNT-based nanobiosensors for in vivo use, in this case for glucose.^116^ To that end, in this paper, we define fluorescent nanobiosensors as optical nanoscale engineered devices that indicate the presence or absence, or concentration, of an analyte of interest in a living multicellular organism through a modulation of transducer signal (plants or animals). In this review, we specifically focused on those using the SWCNT as a transducer.

In vivo sensor applications require sensor confinement to a particular area within the organism to prevent diminution of the signal due to sensor diffusion. It also prevents nonspecific detection within cells or other body compartments, uptake by macrophages or other endocytic cells, biofouling, and/or other conditions that would prevent ideal sensing. Here, we detail the four methods used thus far for sensor deployment in vivo: systemic or local direct injection; encapsulation within a semipermeable membrane; encapsulation within a preformed hydrogel implant device; and injection of a in situ-gelling hydrogel-SWCNT hybrid depot material. In this section, we further consider exogenous (hardware or software, non-nanotube) methodologies designed to improve in vivo signal collection and finally consider the important question of SWCNT safety in live animals and plants.

### Direct Injection or Infiltration of SWCNT Either Systemically or Locally

**Figure 3:**
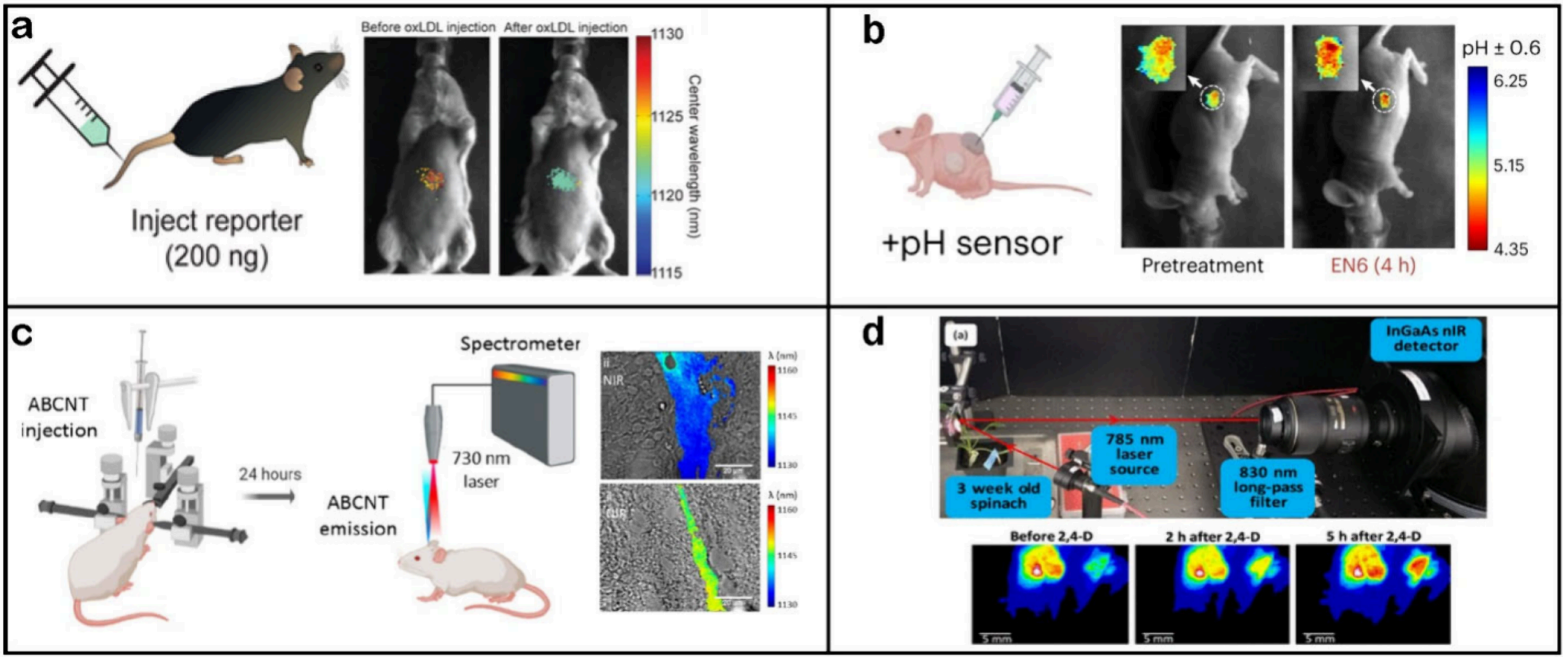
Examples of direct SWCNT sensor application. A) A fluorescent SWCNT sensor was injected intravenously, exhibited liver accumulation and imaged with a near-infrared hyperspectral microscope, where it detected lipid accumulation.^117^ Reprinted in part with permission from Galassi et al. 2018 *Sci. Trans. Med*. copyright AAAS. B) A SWCNT sensor designed to detect pH was injected intratumorally into mice with an ovarian cancer xenograft.^104^ Reproduced in part with permission from Kim et al. 2023 *Nat. Chem. Biol.* copyright Springer Nature. C) A fluorescent SWCNT sensor designed to detect the Alzheimer’s disease protein amyloid-beta was injected via a stereotactic device intracranially into the hippocampus of a mouse disease model.^119^ Reproduced in part with permission from Antman-Passig et al. 2022 *ACS Nano* copyright American Chemical Society. D) A SWCNT-based sensor designed to detect synthetic plant hormones auxins was added to plant leaves and was imaged in the near-infrared region.^90^ Reproduced in part with permission from Ang et al. 2021 *ACS Sensors* copyright American Chemical Society.

To deploy sensors in vivo, a few approaches have used direct compartmental injection, allowing access to only the site of interest for sensing (Figure 3). The most straightforward of these methods has used intravenous injection into mice, allowing SWCNT-based sensors to circulate through the vasculature and ultimately accumulate in the Kupffer cells of the liver.^117,118^ A similar strategy has used intratumor injection into mice with ovarian cancer xenografts.^104^ A stereotactic device has been used for intracranial SWCNT injection into specific compartments of the brain, including the hippocampus.^119,120^ An analogous method has been demonstrated in plants, where SWCNT sensor formulations were applied directly to plant leaves or roots and allowed to diffuse into the tissue.^90^ All instances of in planta SWCNT-based detection have used this method.^30,121−124^ A substantial advantage of these approaches is that they allow simple direct injection/application with no invasiveness, though the SWCNT may diffuse from the site of interest over time or the signal become degraded. Thus, in many cases, these diagnostics are transient and limited to detection in the cell and/or tissue compartment in which the sensor localizes. These methods have proven useful for studying preclinical disease models and accumulation of toxins in plants, though they may be more difficult to translate to the clinic or field due to diffusion, degradation, or depth of signal penetration.

### Encapsulation of SWCNT within a Semipermeable Membrane Device

**Figure 4:**
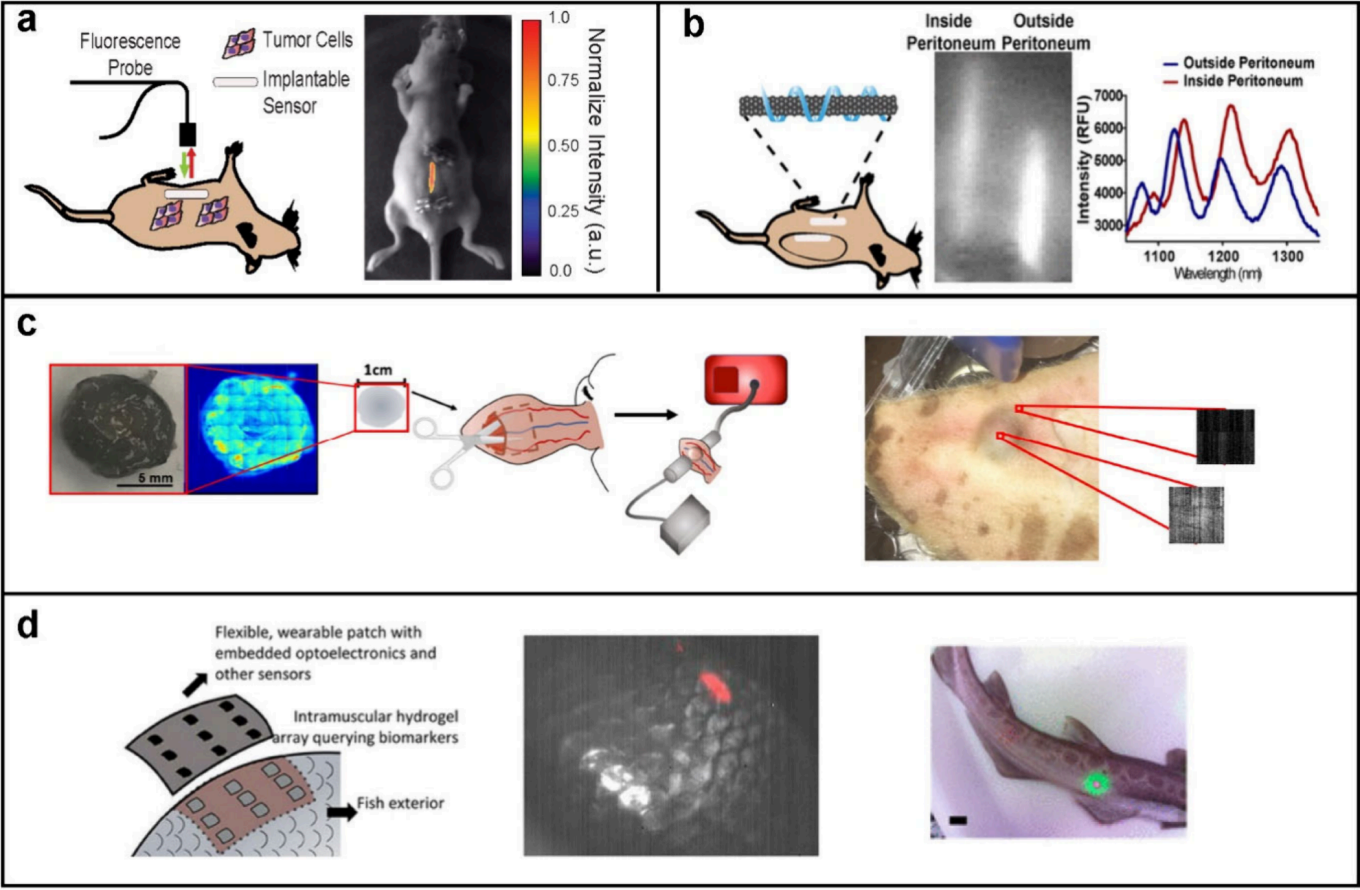
Examples of encapsulated SWCNT application. A) A SWCNT sensor for the ovarian cancer protein biomarker HE4 was encapsulated inside a semipermeable dialysis membrane surgically implanted into mice with orthotopic ovarian cancer xenografts.^21^ Reproduced in part with permission from Williams et al. 2018 *Sci. Adv.* copyright AAAS. B) A SWCNT sensor was used to detect doxorubicin in mice after embedding it within semipermeable dialysis membranes and implantation into the peritoneal cavity or subcutaneously in mice.^125^ Reproduced in part with permission from Harvey et al. 2019 *Nano Lett.* copyright American Chemical Society. C) A preformed hydrogel composed of alginate and embedded with SWCNT sensors for nitric oxide were implanted into mice (not shown) and the ears of sheep.^137^ Reprinted in part with permission from Hofferber et al. 2022 *Nanomed.: Nanotech., Bio., Med.* copyright Elsevier. D) A polyethylene glycol diacrylate (PEGDA) hydrogel array with riboflavin-responsive SWCNT sensors was embedded beneath the scales of several species of fishes.^130^ Reproduced in part with permission from Lee et al. 2019 *ACS Sensors* copyright American Chemical Society.

One strategy for SWCNT sequestration and biofouling prevention is sensor encapsulation within a semipermeable dialysis membrane (Figure 4). The first such examples embedded various SWCNT-based sensors into a manufactured, biocompatible dialysis membrane with a 500 kDa molecular weight cutoff.^21,80,125^ The authors calculated that SWCNT sensor constructs were typically larger than this size, preventing sensor diffusion out of the device, but allowing diffusion of smaller analytes into the device. Those studies detected analytes ranging from small molecules to nucleic acids and proteins, demonstrating wide applicability. However, this method required minor surgery to implant the device subcutaneously and into the peritoneal cavity, as it used a semirigid device membrane. Significant advantages of this method were that it allowed for simple filling of the membrane with a small volume (<10 μL) of solution-phase SWCNT—the same environment in which typical testing is performed—and the sealed membrane was stable for over a month. While this device itself was not evaluated for safety, a commercially produced biocompatible PVDF membrane used in other in vivo applications was chosen as the outer implant device. This type of device may be valuable in the future if secured to another implanted device or fastened to tissue inside a patient.

### Encapsulation of SWCNT within a Preformed Hydrogel Implant

The original and most commonly used method for nanotube sensor in vivo deployment has been encapsulation within a polymeric hydrogel implant.^126^ The first such implanted nanotube sensor was with a nitric oxide (NO) sensor embedded within an alginate hydrogel.^118^ This hydrogel-based device was surgically implanted subcutaneously and impressively demonstrated a bright fluorescence up to 300 days. However, this method also required minor surgery for implantation of the preformed gel. This platform was further explored for use in the ears of sheep.^127^

A more recent iteration of preformed hydrogel implants with embedded SWCNT sensors is with polyethylene glycol diacrylate (PEGDA) gels.^91,128−131^ These sensor-PEGDA constructs have generally similar benefits and drawbacks as the alginate device, although perhaps with greater ease of use and tunability. One study investigated in-depth the effects of sensor-PEGDA cross-linking density on inflammatory responses in vivo, evaluating several different SWCNT preparations.^132^ That study determined that local inflammation was resolved after one month; however, acute inflammation was highly dependent upon hydrogel porosity and SWCNT wrapping.^132^ One study even encapsulated the SWCNT-PEGDA hydrogel inside of a semipermeable membrane, finding reduced biofouling and improved sensitivity.^91^ Interestingly, PEGDA hydrogel-SWCNT complexes have been used in several animals beyond mice, including fishes and eels.^130^

There is substantial ongoing work to develop additional hydrogel systems for SWCNT encapsulation and implantation as well as exciting in vitro applications. For example, hyaluronic acid, chitosan, and alginate-acrylamide hydrogel-based devices have also been explored, but only in vitro thus far.^133,134^ A Fmoc-diphenylalanine hydrogel system has been deployed in engineered tissue models.^135^ A separate spin-coated SWCNT-hydrogel formulation was used to create an on-board bandage-style sensor made of alginate.^136^ Though preformed gels do require some minimal level of invasiveness for surgical implantation, they do allow for a modular form factor and well-controlled pore size of the gel. It should be noted that to date, hydrogel-based in vivo sensor development has only been demonstrated for small molecule analytes, though it is likely that tunable gels do not limit the size of the analyte.

### Direct Injection of an in Situ-Gelling Hydrogel SWCNT Hybrid

Our lab recently sought to overcome some of the hurdles above through the development of an injectable hydrogel-based SWCNT system.^134^ This study evaluated several versions of a methacrylated methylcellulose-based hydrogel, including sulfonated and nonsulfonated versions at various polymer concentrations. Both thermal gelation and redox-initiated gelation were investigated. Redox-initiated gelation is achieved through use of a dual-barrel syringe, allowing redox initiators and the methylcellulose polymer to mix only upon coextrusion from the syringe. The fluorescence of SWCNTs injected in this manner was followed for 2 months in mice and found to be stable and bright. This study investigated the detection of the small molecule chemotherapeutic doxorubicin in vivo, though in vitro studies were successfully conducted with larger biological analytes, such as proteins and smaller ions. The benefits of this system are the tunability of hydrogel systems with no requirement for surgical implantation—just injection with a dual-barrel syringe. This system solidified within 15 min with minimal diffusion from the subcutaneous injection site due to viscosity, though it should be noted it is space-filling, which may not be beneficial in all circumstances. In addition, safety studies on this SWCNT-hydrogel formulation have not yet been performed; however, the studies did use a modified methylcellulose macromer previous used for soft tissue bulking applications in vivo with excellent biocompatibility and stability over time.^138,139^ We anticipate that this may be useful for site-specific diagnostics or in combination with tissue-filling biomaterials.

### Enhancement of In Vivo SWCNT Fluorescence Signal Processing

In vivo nanosensor measurements have been obtained through both spectral and image-based techniques. Initial applications used an in vivo imaging system built for fluorophores with emission wavelengths up to 1050 nm (a charge-coupled device, or CCD, camera).^118^ Typical SWCNT imaging in the NIR uses an InGaAs camera or array for short-wave infrared applications.^114,140^ Several studies have used an InGaAs array coupled to laser excitation with a point-and-shoot fiber optic device to collect data in a more versatile and semimobile format.^21,80,117,134^ An additional advancement was made in the development of NIR hyperspectral whole-animal imaging, which allowed for simultaneous spectral and spatial information in live animals.^21,117,141^ While these measurements devices have been useful in the lab, a field-deployable system based on Raspberry Pi computers and cameras has been developed to reduce both cost and weight of common equipment.^90,123,124^

Further methods of in vivo signal enhancement will also increase the nanosensor applicability. For example, several studies have developed and used a wavelength-induced frequency filtering (WIFF) method, allowing for an enhanced signal-to-noise ratio.^129^ In brief, the technique employs three excitation lasers (instead of just one) to oscillate the excitation wavelength about the sensor’s absorption wavelength at a fixed frequency. This enabled interrogation of sensors implanted 5.5 cm below the surface of chicken breast ex vivo, compared to just 3.2 cm without WIFF. Another methodology for enhanced in vivo signal accuracy is spectral triangulation.^142^ In this method, Matrigel-encapsulated SWCNTs were coregistered with computed tomography (CT) and validated by magnetic resonance imaging (MRI) following excitation by an LED matrix array instead of a single-point laser. Further data processing and equipment advancements are likely and will push SWCNT-based sensors closer to clinical utility or other commercial applications by improving signal strength, signal processing, penetration depth, and sensitivity.

### Safety of SWCNT In Vivo

There have been several studies and substantial debate regarding the safety of SWCNTs.^143,144^ Clearly, this is of paramount focus for any translational, patient-focused application or even for use in animal disease models or other environmental applications. There have been several studies that generically claimed SWCNT are toxic to humans, though those broad claims were based on narrow data sets.^143,144^ Given that carbon nanotubes in general, and even SWCNTs specifically, encompass a broad class of materials with many possible functionalization schemes and methods of introduction to the body, their safety is heavily context-dependent^144^—as is that of any medicine, diagnostic, food, or other commonly encountered material. Substantial investigation has gone into understanding the interface between SWCNTs and biological components in vivo, especially concerning protein binding and corona formation.^3,145,146^ For example, corona formation with common serum or plant proteins is known to improve the biocompatibility of these materials.^3,147^ And thus, with an improved understanding of protein corona dynamics and materials design considerations driving such formation, it is possible to design mitigation strategies for issues related to SWCNT safety.^148^ Indeed, none of the applications reported any side effects of in vivo SWCNT-based sensing, with most performing some level of safety analysis. Specific safety studies have demonstrated that well-dispersed polymer- (or nucleic acid-) coated SWCNTs, as are required to make nanosensors, are biocompatible with no toxic effects in mice and nonhuman primates.^114,141,149^ Studies in plants have also demonstrated no detrimental effects after SWCNT sensor applications.^150^ That being said, as with all translational devices, safety and toxicology should be specifically studied for each device, including cytotoxicity, immune reactivity, pharmacology (if any), biofouling, potential for bacterial infection, and environmental impacts.^144^

#### Small Molecule Analytes Which Have Been Detected by SWCNT In Vivo

As described above, the detection of glucose and other small-molecule analytes in vivo with SWCNTs has been a goal for at least two decades.^116^ In vivo or onboard glucose detection has been a broader goal of the biosensor community for longer, and has recently been realized with other technologies, namely electrochemical-based CGM.^15^ Glucose, and other small molecule analytes such as dopamine and NO, have been detected ex vivo in rodent brain slices and other biological tissues/fluids.^106,107^

### Nitric Oxide (NO) and H_2_O_2_, Free Radicals

CoPhMoRe-like sensors were originally developed and demonstrated to detect the free radical nitric oxide in vivo via PEGylated oligonucleotide SWCNTs that were embedded in a preformed hydrogel.^118^ In vitro, the sensor was shown to be the best quenched by NO of the 10 SWCNT constructs tested.^87^ Screening of the sensor against 35 other biological molecules, including 9 other reactive oxygen species, confirmed selectivity. An implant was created by encapsulating this sensor in an alginate hydrogel, which was found to preserve sensitivity and selectivity.^87^ When subcutaneously implanted in a mouse, the sensor’s fluorescence totally quenched within 20 min and then slowly recovered over 4 days. The authors attribute this quenching to NO produced in the wound-healing process.^151^ Significantly, a long-term study of the implant found that it continued to fluoresce for 300 days postimplantation, and caused no inflammation of the surrounding tissue.^118^ In addition, though not strictly in vivo, an on-board bandage-style device was designed to detect free radicals secreted from the skin.^136^ In this design, a fluorescent SWCNT sensor was electrospun into a polymer microfiber, which was later incorporated into a commercial bandage.

Separately, a ratiometric SWCNT-based sensor design for NO was used in *Arabidopsis thaliana* leaves.^152^ The sensors were added directly to the leaves and allowed to infiltrate, wherein they were imaged with a near-infrared camera. Ratiometric intensity changes were evaluated to determine the presence of NO in these leaves. In the same study, a distinct ratiometric sensor was described for hydrogen peroxide (H_2_O_2_) and similarly imaged in the leaves of the same plant. This work was also extended in a separate study in which in vivo remote monitoring of SWCNT sensors was used to evaluate plant stressors such as UV-B light and pathogen-produced peptides.^150^ Excitingly, no negative effects were observed on the plant as a result of the sensor application. A similar sensor design was used to detect a H_2_O_2_ signaling in real-time after induced injury in lettuce, arugula, spinach, strawberry blite, sorrel, and *Arabadopsis*.^122^ Beyond the applications for monitoring plant health, this study investigated the biology of plant receptor channels that propagate hydrogen peroxide signaling in real time.

The first study to image SWCNT-based sensors in a large mammal model, namely sheep, recently evaluated another version of this implant.^153^ The implant described in this work used a different oligonucleotide sequence and alginate gel preparation and was tested in the ears of 14 sheep. One-week postimplantation, sensor fluorescence was detected in 9 out of 14 sheep. A method for SWCNT extraction and quantification recovered up to 90% of SWCNTs with none detected in major organs.^127^ These results are consistent with earlier in vitro studies of similar implants, which also found that the gels effectively sequestered the sensors.^154^

### Gibberellins, a Class of Plant Growth Hormones

A CoPhMoRe-based approach with amphiphilic polymers was employed to develop a sensor for plant growth hormones of the gibberellin family.^121^ Gibberellins regulate plant growth and are responsive to local environmental factors such as drought, salinity, toxins, or pathogens.^155^ Due to the similarity of gibberellin family structures, they are difficult to distinguish and reliably detect in a nondestructive manner. After screening the polymer library against various gibberellins and closely related molecules, sensors that could detect GA_3_ and GA_4_ were obtained and deployed in *Arabidopsis* plants, lettuce, and basil roots by direct application and infiltration into the root. As the SWCNT response was intensity-based, an innovative reference signal strategy was devised to benchmark fluorescence to invariant Raman G-band intensity. This necessitated innovation of a dual Raman/NIR fluorimeter, which allowed for spatiotemporal detection of the targets in root systems as they develop. This strategy was successful in both nonmodel plants as well as model plants that did or did not overexpress GA_3_.

### Picric Acid, a Nitroaromatic Explosive

Prior studies had developed a peptide-based SWCNT chaperone sensor that had high selectivity for several nitroaromatic explosives.^63^ Bombolitin II, an amphiphilic peptide derived from bumblebee venom, was adsorbed to the surface of SWCNTs, solubilizing and functionalizing them. This complex was screened and found to be responsive to RDX and picric acid and others to a lesser extent. Nitroaromatic explosives are obvious threats to safety, and it is well-known that plants sample and concentrate such molecules from the groundwater and air.^156^ Therefore, a nanobionic autosampling plant detector for explosives detection was developed using the SWCNT-based sensor. This sensor was continuously monitored by an external detector composed of a Raspberry Pi processor and charge-coupled device (CCD) camera, similar to standard smartphones, allowing external monitoring of explosive levels or so-called standoff detection. This setup was able to monitor time-dependent infiltration and the concentration of picric acid into spinach leaves, marking a low-cost alternative for standoff detection of environmental explosives.

### Arsenic, a Toxic Heavy Metal Pollutant

A library of ssDNA-SWCNT complexes were screened for their ability to respond to arsenite (As^3+^) and further used in a nanobionic plant-based autosampler similarly to as above for picric acid.^123^ Arsenic is a toxic heavy metal often found in pesticides and produced as waste products from human industrial processes.^157^ Substantial accumulate of arsenic in the groundwater and in crops poses a threat to both human health and natural systems.^158^ However, remediation is possible when arsenic is detected before it becomes a health or environmental concern. Therefore, a nanobionic plant sensor for arsenic was designed to measure trace amounts in the soil and groundwater after uptake by spinach plants. The SWCNT sensor was measured by a stand-off detector 1 m away, which originally detected a 10 μM solution of arsenite added to the leaf. Longer-term studies found that the sensor was able to detect 0.2 ppb arsenite 2 weeks after exposure—demonstrating the extremely sensitive and potential long-term use nature of the sensor. The sensor was then applied to use in ferns, which are known to tolerate and hyperaccumulate high arsenic levels, as a means of understanding the mechanisms of this uptake pathway.

### Doxorubicin, an Anthracycline Chemotherapeutic

A noncomplementary oligonucleotide-SWCNT sensor within a semipermeable membrane was developed that is capable of detecting the small molecule anthracycline doxorubicin (DOX) when implanted in a murine model.^159^ DOX is an effective and broad-spectrum chemotherapeutic used to treat many cancers, including breast, lung, bladder, and others.^160^ While monitoring is desirable for all chemotherapeutics, it would be especially valuable to monitor lifetime DOX exposure since the drug exhibits cumulative cardiotoxicity.^161,162^ Chemotherapeutic monitoring is often difficult since a full understanding of systemic exposure necessitates multiple blood samples, and is not currently possible for agents with prohibitively short half-lives.^9^ A sensor implant could circumnavigate all of these challenges, enabling clinicians to tailor treatment regimens. Noncomplementary DOX detection is possible since DOX is highly conjugated, and so has high affinity for the SWCNT surface.^159^ Composed of oligonucleotide-SWCNTs contained in a semipermeable dialysis membrane, the implant responded to DOX (quenching and red-shifting) at concentrations as low as 0.5 μM in vitro in an irreversible and cumulative manner. An irreversible, cumulative response is desirable, as the toxicity of DOX is itself irreversible and cumulative; a reversible sensor would not report on lifetime exposure. Surgically implanted into the peritoneal cavities of live mice, this device discriminated between those injected with buffer and those injected with 50 nmol DOX. The device also demonstrated compartmental detection and may allow for segmented pharmacological analyses, which is useful since DOX is sometimes administered in situ.^163^

A second method of doxorubicin detection using a noninvasive, injectable hydrogel implant was recently demonstrated.^134^ This device utilized a previously demonstrated, biocompatible hydrogel based on a derivative of methylcellulose.^138^ The sensor-containing polymer solution was injected from a dual barrel syringe fitted with a mixing tip. Either barrel of the syringe contained one of the two cross-linking agents (ascorbic acid and ammonium persulfate), which began rapid polymerization postinjection. This injectable, noninvasive format represents a significant step toward clinical translation by circumventing the need for surgical implantation.

### Riboflavin and Ascorbic Acid, Vitamins

CoPhMoRe SWCNT-based sensors encapsulated within preformed hydrogels have also demonstrated transcutaneous detection of small molecules riboflavin and ascorbate in mice.^128^ Whereas the ascorbate sensor was found to respond reversibly, the riboflavin sensor responded irreversibly. For better performance in vivo, a ratiometric implant incorporating a desensitized reference SWCNT was devised. These sensors were encapsulated into poly(ethylene glycol) diacrylate (PEGDA) hydrogels alongside reference SWCNTs to form implantable devices. The reference SWCNTs were prepared by wrapping SWCNTs with a styrene polymer shown to effectively passivate them against analyte interaction. It was necessary to incubate these devices in water for 2 days prior to use, as they were found to leach significant sensor content during this time, but not afterward. These sensors enabled discrimination between mice intraperitoneally injected with analytes and those injected with controls. Three days after implantation, these devices were removed and demonstrated continued functionality in vitro.

The WIFF (wavelength-induced frequency filtering) technique to improve the signal-to-noise ratio was demonstrated with this riboflavin sensor in vivo.^129^ Using WIFF, the riboflavin sensor could be interrogated at depths of approximately 1.5 cm in living mice, corresponding to the path length of light through the entire animal. This was not possible with a typical single-laser excitation. WIFF also decreased variance (n = 5) by almost an order of magnitude (from 9% to 1%). This enabled the riboflavin concentration to be calculated from the sensor signal in vivo. Some of this decrease in variance may be attributable to WIFF’s negation of signal drift due to implant movement, found to be as high as 20% over 10 min without WIFF.

A similar preformed hydrogel was also tolerated by marine organisms.^130^ After calibrating the sensor in vitro and optimizing implant depth ex vivo, the implant was injected into a European eel (*Anguilla anguilla*), an eastern river cooter (*Pseudemmys concinna*), a catshark (*Scyliorhinus stellaris*), and a goldfish (*Carassius auratus*). While high-resolution ultrasound confirmed that the implants were tolerated by the eel and catshark, the turtle’s injection site had not healed 33 days postimplantation. Upon extraction from the turtle, the implant was found to be covered by tissue, which is indicative of an undesirable foreign body response. However, the authors noted that the irritation may have been provoked by an infection rather than the implant itself. Attempts were made to image the implants but they were unsuccessful because of animal movement and low excitation power. Behavioral analysis of the experimental goldfish versus the control goldfish confirmed implant tolerance.

### Temozolomide, a Chemotherapeutic, and Its Metabolite

A more recent CoPhMoRe-based SWCNT sensor used WIFF for detection of a metabolite of the small molecule chemotherapeutic Temozolomide in vivo using a preformed hydrogel.^131^ The sensor was employed to detect 5-aminomidazole-4-carboxamide (AIC) in vivo, a metabolite of pro-drug Temozolomide (TMZ), the standard glioblastoma chemotherapeutic since at least 2005.^131,164,165^ Ten oligonucleotide-SWCNT complexes were screened against four chemotherapeutics, revealing a prominent intensity increase response to TMZ and AIC for one complex.^131^ For in vivo experiments, an implantable device was created by encapsulating the sensor in a PEGDA hydrogel.^131^ Hydrogel encapsulation enabled the sensor to function in serum without its response magnitude appreciably diminishing, though the response kinetics were slower. After confirming the function in cell culture, the device was implanted in mice and monitored during TMZ injection. The implanted devices showed fluorescence responses consistent with the TMZ metabolization kinetics. Use of calibration curves allowed the response of these sensors to be converted to AIC concentrations. The WIFF approach was also shown to enable interrogation of this sensor implanted 2.4 cm deep in the brain of a porcine fetus, a feat not possible by traditional single-laser measurements without intracranial fiber optics or windows.^129,131^

### Progesterone, a Hormone

A variation of CoPhMoRe, using surfactant templating, has recently been applied to develop sensors with better affinity for desired analytes, with demonstrated progesterone and cortisol sensing in vivo.^91^ These sensors were embedded in a preformed hydrogel, which was subsequently placed in a dialysis membrane. Analogous to molecularly imprinted polymer (MIP)-based sensors, these devices were fabricated using surfactants containing a template moiety that adsorbs to the SWCNT surface. In the presence of the analyte, this template desorbs from the SWCNT surface and is replaced by the analyte. This is different from MIP-based sensors, which are typically templated with analyte molecules later removed from a polymer layer in a wash step, leaving behind binding pockets sterically and electrostatically conducive to analyte-binding.^166^ Whereas analyte binding modulates the signal for MIP-based sensors, analyte-mediated template displacement modulates the signal for these sensors.

After screening a library of 80 acrylated cortisol-templated polymer-SWCNT combinations against 11 steroids, potential sensors for progesterone and cortisol emerged with good sensitivity and moderate selectivity.^91^ Sensor and control implants were constructed by encapsulating progesterone sensors and styrene-passivated SWCNTs respectively in polyethylene glycol hydrogels in dialysis membranes. Sensor function was tested in live mice by comparing the postimplantation fluorescence of sensors previously incubated with 100 μM progesterone to those previously incubated in a buffer solution as a control (n = 3). The progesterone-incubated implant showed changes in fluorescence as progesterone diffused out of the membrane. These changes were not seen for the control implant, confirming sensor operation in vivo. Tissue samples taken from implant regions 28 days later showed signs of normal wound healing, demonstrating biocompatibility. Implants with the dialysis membrane responded to progesterone for 24 h, compared to 2 h with no membrane. FTIR spectra of implants without membrane encapsulation showed no significant changes postimplantation, indicating that chemical modification of the bulk gel had not occurred. This led the authors to speculate that the hydrogel’s pores became clogged (preventing the analyte from interacting with the sensor), or else that the binding site might have been rendered nonfunctional by chemical modification or the irreversible binding of some interferant.

### Auxins, Synthetic Plant Hormones

A guided screen approach has also been used to develop CoPhMoRe SWCNT sensors for the detection of synthetic small molecule auxins used as herbicides in plants.^90^ These sensors were applied directly to plant leaves and subsequently infiltrated into the tissue. In excess, auxins pose a serious toxicity risk to the environment and the individual; a better understanding of their role in botanical signaling pathways would facilitate a more responsible use of these chemicals.^167,168^ The sensor was designed by screening six polymer-SWCNT constructs against a library of 12 plant hormones. The polymers selected for this screening were already known to bind auxin carboxylates in the absence of SWCNTs. SWCNT constructs showed a selective response to four auxins, although two of these could only be detected at concentrations above their relevant range. Ultimately, two quenching-based sensors demonstrated good selectivity: one for naphthalene acetic acid (NAA) and one for 2,4-dichlorophenoxyacetic acid (2,4-D). Both chemicals are routinely used in agriculture, although there is growing concern over the toxicity of 2,4-D.

A ratiometric design, incorporating both sensor and reference SWCNTs, was used to test these devices in whole, intact spinach plants.^90^ The plants showed no adverse response to sensor infiltration during the entire four week observation period. These sensors were quenched when the plants were dosed with their analytes, confirming the in vivo function. Continuous monitoring of these sensors revealed the rate of analyte metabolism. The study found that NAA was metabolized faster than 2,4-D, making the latter a more effective herbicide. The 2,4-D sensor was also used to gauge the susceptibility of pak choy and rice to 2,4-D, since plants that metabolize it faster are able to mitigate its effects.^169,170^

### Lysosomal pH

SWCNT-based sensors have additionally been demonstrated in vivo to detect lysosomal pH after intratumoral injection into ovarian cancer xenografts.^104^ In this work, SWCNTs were functionalized with sp^3^ defects to create organic color centers (OCCs) that were wrapped with noncomplementary DNA. OCC-SWCNT sensors demonstrated a monotonic change in wavelength in vitro in response to buffer pH modulation. They responded reversibly to pharmacological lysosomal pH alteration in several cancer and noncancer cell lines in vitro. Nude mice were implanted with SKOV3 ovarian cancer cells via flank xenograft and subsequently treated with an autophagy modulator, EN6, which caused lysosomal hyperacidification. The intratumorally injected nanosensors were responsive to subtle pH changes, revealing that the sensor responded sensitively to autophagy in vivo.

#### Biological Macromolecule Analytes That Have Been Detected by SWCNTs In Vivo

Beyond small molecule detection, many larger biological molecules have established diagnostic and prognostic profiles for disease detection and monitoring. The first two examples here did not demonstrate spectroscopic-based detection in vivo, though imaging-based methods were used to detect specific molecular analytes of interest through an increase of signal brightness. The latter examples primarily used spectral methodology to quantify the biomarkers of interest.

##### PSMA, a Surface Protein on LNCaP Prostate Cancer Cells

An intensity-based sensor for the presence prostate cancer cell line LNCaP was demonstrated in live mice bearing a flank xenograft.^171^ M13 bacteriophage were engineered to display an antibody against prostate-specific membrane antigen (PSMA) and a protein (p8) that binds well to SWCNTs, a multifunctional scaffold designed in previous studies.^172,173^ Those sensor constructs or controls were injected intravenously into mice bearing flank LNCaP tumors expressing PSMA. Near-infrared fluorescence imaging was used to evaluate the presence of these tumors, demonstrating the first cell/protein-specific SWCNT sensor in vivo.

### *Staphylococcus aureus* Bacterial Surface Proteins

A sensor for *Staphylococcus aureus* Gram-positive bacteria was developed to detect bacterial infections in vivo.^174^ Similar to above, M13 bacteriophage were modified with targeting antibodies for *S. aureus* and a protein (p8) that binds to SWCNTs. This construct resulted in well-dispersed M13-SWCNT sensor constructs that were subsequently injected intravenously into mice with *S. aureus, E. coli*, or control. The animals were then imaged, and the brightness was determined at the site of infection, wherein the M13-SWCNT was significantly selective for the *S. aureus* infection. While it was shown that the majority of the sensor construct localized to the liver, the second-highest site of localization was the infection site.

### microRNA mIR-19

A sensor for the microRNA miR-19, known to play a role in oncogenesis,^175^ was designed based on complementary base-pairing and deployed in vivo using a dialysis membrane implant.^80^ This sensor consisted of a SWCNT wrapped by a heterobifunctional oligonucleotide possessing one domain for SWCNT adhesion and another for base-pairing with miR-19.^80^ The recognition domain is unwrapped from the SWCNT surface upon miR-19 pairing, modulating SWCNT fluorescence. The magnitude of this modulation is increased in the presence of SDBS, thought to replace the recognition domain on the SWCNT surface upon the complexation of miR-19 with the recognition domain. Because the recognition domain is the complement of miR-19, the sensor exhibits very good specificity for miR-19 compared with other oligonucleotides. An implant was created by loading the sensor construct and SDBS into a dialysis membrane (molecular weight cutoff of 500 kDa). Surgically implanted into the peritoneal cavities of mice, this device responded to injections of miR-19 in amounts as low as 100 pmol.

### HE4, an Ovarian Cancer Protein Biomarker

While several antibody (Ab) SWCNT sensors have been described,^22^ one has been demonstrated in vivo.^21^ The target analyte of this device was ovarian cancer biomarker human epididymis protein 4 (HE4), which was detected in live mice following implantation within a dialysis membrane device. After Ab conjugation to oligonucleotide-wrapped SWCNTs, the Ab-SWCNT complex was passivated to interferants by incubation with bovine serum albumin (BSA), a previously demonstrated approach for increasing specificity by occluding potential nonspecific (off-target) binding sites.^22^ After these HE4 sensors were demonstrated to discriminate high-grade serous ovarian carcinoma patient sera and ascites from benign or noncancerous controls, they were used to detect ovarian cancer in mice.^21^ An implant of Ab-SWCNT sensors was created in a semipermeable dialysis membrane bag (500 kDa molecular weight cutoff). In vivo experiments were performed by surgically implanting these devices into the peritoneal cavities of mice. Noninvasive fluorescence excitation and emission measurements of these implants were shown to discriminate between mice injected with 10 pmol of HE4 and those injected with BSA as a control. Further, the implant was shown to discriminate between mice implanted with HE4-producing and non-HE4-producing tumors. Device function in vivo was confirmed for up to 24 h, and emission intensity was noted to be consistent across a 38-day period.

### Lipids as Indicators of Lysosomal Storage Disorders

A noncomplementary DNA-SWCNT sensor for lipid storage disorders has been demonstrated in vivo via direct injection and subsequent liver accumulation,^117^ utilizing the inherent affinity of SWCNTs for hydrophobic fatty acids^176^ and lipid derivatives.^177^ Though it is more common to confine SWCNT-based sensors to an implant, avoiding cellular uptake and protein interference, they have also been administered intravenously to accumulate in liver macrophages^141^ for disease monitoring.^117^ This approach was used to distinguish healthy mice in murine models of diseases characterized by endolysosomal lipid accumulation: nonalcoholic fatty liver disease, nonalcoholic steatohepatitis, and Niemann-Pick disease (types A/B and C).^117^ This study used a sensor composed of monochiral DNA-SWCNTs. Sensor fluorescence was modulated by cholesterol or sphingomyelin, known to be overexpressed in these diseases.^178,179^ After the sensor was confirmed to respond to water-soluble lipid analogs in silico, in vitro, and in cell culture,^180^ it was intravenously administered to mice in doses of 200 ng.^117^ These sensors were imaged 24 h postinjection. The study also used these sensors to successfully monitor the uptake and accumulation of intravenously administered oxidized low-density lipoprotein, thought to be involved in atherosclerosis.^181^

In another application of this sensor, it was directly injected into the hippocampus of a mouse model of Niemann-Pick type A disease.^120^ This model exhibits increased lysosomal accumulation of sphingomyelin, which was detected through the skull of mice compared with healthy controls. The sensor demonstrated a shift in the center wavelength in vivo and in tissue slices ex vivo. It should be noted that no toxicity from this sensor was found in the hippocampus of these animals.

### Amyloid-beta, a Biomarker of Alzheimer’s Disease

A surfactant-based sensor with self-assembled amyloid-beta protein has also been demonstrated for use in vivo following intracranial injection into a mouse disease model.^119^ The sensor was constructed through SWCNT dispersion with sodium deoxycholate and subsequent self-assembly by adsorption of the Alzheimer’s-related protein amyloid beta 42 (Aβ42), with the sensing mechanism taking advantage of the fact that Aβ proteins readily form aggregates. The function of this sensor was demonstrated using a cell line model of Alzheimer’s disease in vitro with strong selectivity for Aβ_42_. It was then intracranially injected with a stereotactic device into the hippocampus of the 5XFAD genetic mouse model of Alzheimer’s disease. The sensor was observed in an extracellular pool and demonstrated a distinctive shift in older mice with advanced disease but not in younger mice with fewer Aβ aggregates, which was confirmed by histology.

#### Conclusions and Future Prospects

**Figure 5:**
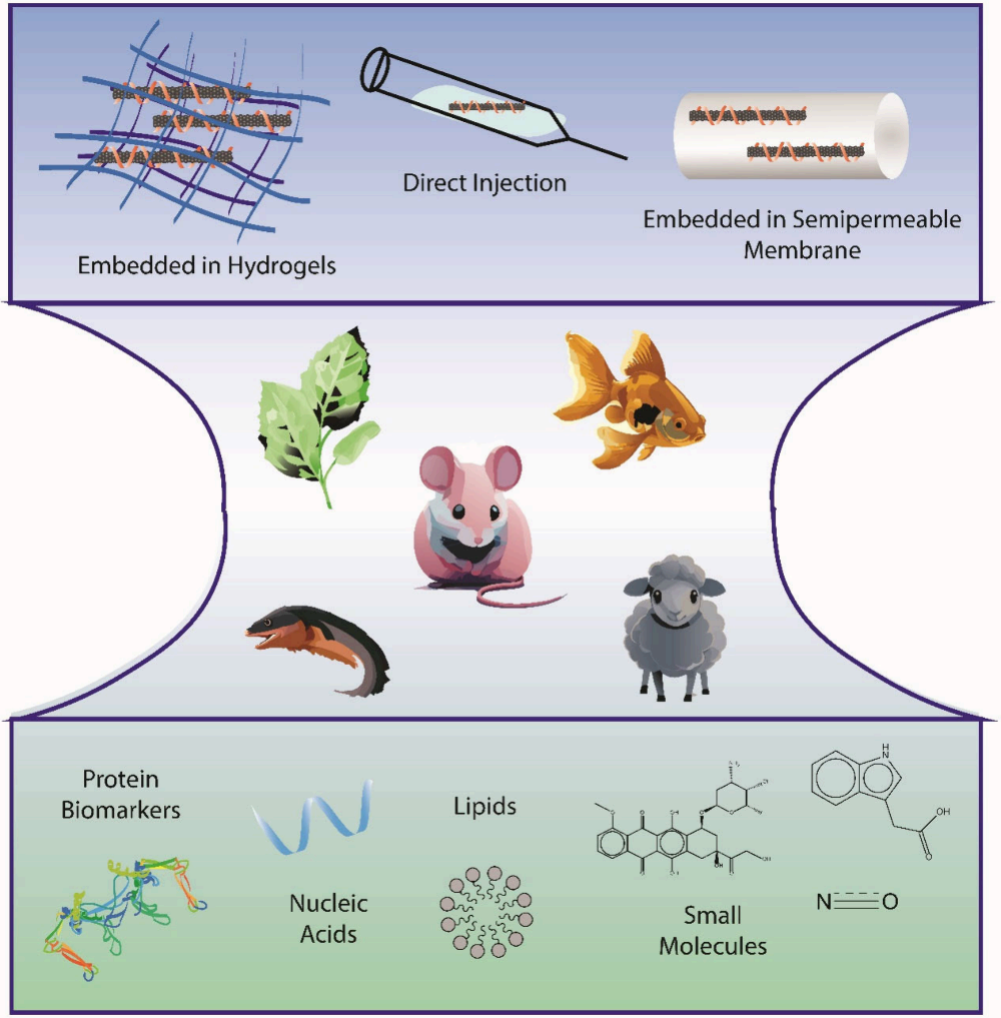
Schematic encompassing common in vivo applications of SWCNT-based sensors. Top represents common methods of in vivo deployment. The middle section represents several different species in which SWCNT sensors have been used, with mice being the most prominent. Bottom demonstrates several various analytes that have been detected in vivo by SWCNT-based sensors.

Because of their optical properties^48,49^ advancing methods of functionalization,^35^ and emerging techniques for deployment in vivo, SWCNTs are promising transducers for in vivo nanobiosensors at depths up to several centimeters.^129,131^ Their use has been demonstrated in this regard in mice, plants, fish, and sheep with in vivo sensitivities often within ranges relevant for field-use sensors or clinical diagnostics (Table 1, Figure 5). Certainly, there are other technologies that have been more well-studied and even translated to clinical use, such as electrochemical sensors for continuous glucose monitoring.^12,182^ To compete with these technologies and even improve upon them, some challenges remain to further SWCNT translation to the clinic or field use.

**Table 1:** Comprehensive Listing of Analytes Detected by SWCNT-Based Optical Nanobiosensors in Living Animals or Plants

Analyte	Recognition Mechanism	Organism(s)	In Vivo Detection Strategy(ies)	In Vivo Detection Level and Sensor Response
*Small Molecule Analytes*				
Nitric oxide (NO)^118,153^ and H_2_O_2_^122,150,152^	CoPhMoRe (DNA + PEG)	Healthy mice, *Arabidopsis thaliana* leaves, and sheep	1) Direct intravenous injection/liver accumulation, 2) direct application to plant leaves/infiltration 3) preformed alginate hydrogel	10–100 μM H_2_O_2_ in *Arabidopsis* leaves (intensity)
Plant hormones gibberellins^121^	CoPhMoRe (amphiphilic polymers)	*Arabidopsis*, lettuce, and basil roots	Direct application to seedlings and roots	100 μM (intensity referenced to Raman G band)
Nitroaromatic explosive picric acid^124^	Bombolitin II amphiphilic peptide	Spinach plants (*Spinacia oleracea*)	Direct infiltration to leaves	0.2 mL solution of 400 μM added to leaves (intensity referenced to nonresponsive SWCNT)
Toxic heavy metal arsenic^123^	CoPhMoRe (ssDNA)	*Pteris cretica* fern, spinach, and rice plants	Direct infiltration to leaves	0.6 and 0.2 ppb after 7 and 14 days (intensity increase compared to reference)
Chemotherapy doxorubicin^134,159^	Noncomplementary DNA	Healthy mice	1) Semipermeable dialysis membrane, 2) injectable methylcellulose hydrogel (first noninvasive detection in mice)	50 nmol (shift and intensity), 330 nmol (shift)
Vitamin riboflavin^128−130^	CoPhMoRe (DNA)	Mice, fetal pig skull (deceased), aquatic species (imaging demonstrated, not sensing)	Preformed PEGDA hydrogel-enhanced by wavelength-induced frequency filtering (WIFF) and field-deployable detectors	30 nmol (intensity)-demonstrated 5.5 μm depth of detection.
TMZ chemotherapy and metabolite AIC^129,131^	CoPhMoRe (DNA)	Mice, fetal pig skull (deceased)	Preformed PEGDA hydrogel-enhanced with WIFF	13 μM in pig skull, 100 μM (intensity)
Steroid progesterone^91^	CoPhMoRe (templated polymer)	Healthy mice	Preformed PEGDA hydrogel plus dialysis membrane	100 μM (intensity)
Auxins NAA and 2,4-D^90^	CoPhMoRe (templated polymer)	Spinach and pak choi plants	Direct leaf application	10–100 μM (intensity)
Lysosomal pH^104^	Noncomplementary organic color center (OCC) SWCNT	Ovarian cancer xenografted mice	Intratumoral injection into ovarian cancer xenografts	Detected pH change in response to 3 authophagy modulators (shift)
*Biological Macromolecule Analytes*				
Prostate cancer cell lines (LNCaP) expressing PSMA^171^	Engineered M13 bacteriophage with anti-PSMA antibody	Mice bearing flank prostate cancer tumors	Direct intravenous injection	Presence of PSMA in 3–7 mm tumors
*Staphylococcus aureus* Gram-positive bacteria^174^	Engineered M13 bacteriophage with anti-*S. aureus* antibody	Mice with *Staph.* infection with *E. coli* infection as control	Direct intravenous injection	Presence of *S. aureus* in the thigh muscle or heart
microRNA miR-19^80^	Complementary DNA	Healthy mice	Dialysis membrane and surgical implantation	100 pmol (shift)
Ovarian cancer protein biomarker HE4^21^	Antibody	Intraperitoneal ovarian cancer mice	Dialysis membrane and surgical implantation	10 pmol (shift) of disease model-expressed HE4
Lipids^117,120^	Noncomplementary DNA	Mice with Niemann-Pick type A	1) Direct intravenous injection/liver accumulation (Kupffer cells), 2) direct stereotactic intracranial injection into the hippocampus	200 μg oxidized LDL, as well as endogenous lipid production in 2 disease models, detection of sphingomyelin in disease model (shift)
Amyloid-beta Alzheimer’s disease protein biomarker^119^	Self-assembly of amyloid-beta on SDC-SWCNT	Alzheimer’s aisease model mice	Stereotactic injection into the hippocampus	Presence of amyloid-beta in transgenic mouse model of Alzheimer’s disease (shift)

While long-term fluorescence has been demonstrated by implanted SWCNTs, sensing has not been demonstrated across a long time frame.^118^ As an alternative to long-term implantation, recovering the sensor after a defined time frame has been demonstrated for hydrogel-encapsulated sensors, though this approach required surgery.^127^ It is therefore necessary to test the sensor lifetime in vivo as foundational knowledge for each individual sensor. For example, sensor evaluation in vivo should be tested for its ability to reliably detect a target over months within live animals or plants to years as a first principle, rather than a saved for downstream testing. With that knowledge, strategies to improve lifetime can be developed pending clinical/environmental goals, including maximizing colloidal stability, minimizing biofouling, or using more stable recognition elements. Another potential barrier to translation is the related concepts of fluorescence depth-of-penetration and signal-to-noise. As it stands, detection of local environments beyond a few centimeters is not possible. This may be overcome by a focus on easy-to-sample environments close to the surface or enhancing detection methodology and quantum yield to sample deeper environments. While also a technology- and engineering-based problem, development and adoption of portable low-cost measurement instrumentation to analyze sensor signal rapidly and in the field is a necessity for broad and equitable adoption. This will include the cooperation of engineers, scientists, industry, capital, and importantly government regulators.

SWCNT-based sensors have substantial potential for clinical diagnostics, disease monitoring, addressing lab-based research questions, and even environmental monitoring. While translation to in vivo studies is just becoming more widespread, along with ex vivo applications, there is substantial potential for innovation and excitement in the field, potentially capitalizing on the promise of using such tools. Future work must strive to build upon the approaches outlined here and work toward improved systems to realize the full potential of the SWCNT.

## Vocabulary

Single-walled carbon nanotube (SWCNT): cylindrical graphitic sp^2^ carbon increasingly used as transducers for in vivo molecular nanbioosensors
Transducers: materials that transmit the present of an analyte to a measurement device through light
Near-infrared or NIR: light in the electromagnetic spectrum from 780–2500 nm
Nanobiosensors: nanoscale engineered devices that indicate the presence or absence, or concentration, of an analyte of interest through a modulation of transducer signal
In vivo: experiments performed in living plants or animals such as mice, fish, or sheep in this paper
Implantable sensor device: a biocompatible sensor that can be placed inside a plant or animal to detect a specific analyte
